# An Improvement in the Method of Determining If Gonorrhoeal Infection Is Preesnt in an Inflamed Prostate Gland or Seminal Vesicle

**Published:** 1908-08

**Authors:** Edgar Ballenger

**Affiliations:** Lecturer on Genito-Urinary Diseases, Atlanta School of Medicine, Atlanta, Ga.


### AN IMPROVEMENT IN THE METHOD OF DETERMINING IF GONORRHOEAL INFECTION IS PRESENT IN AN INFLAMED PROSTATE GLAND OR SEMINAL VESICLE.*

BY EDGAR BALLENGER, M. D., LECTURER ON GENITO-URINARY DISEASES, ATLANTA SCHOOL OF MEDICINE, ATLANTA, GA.

Briefly stated, the method submitted in the following note on ascertaining if gonococci are present is to create a chemical urethral discharge, and when it is well established, to seal the meatus with collodion or adhesive plaster and then to massage the secretion from the prostates of vesicles into the inflamed canal, where it is left until the patient urinates. Gonococci will proliferate if placed in this favorable invironment, and may be demonstrated in the subsequent discharge.

The importance of knowing whether or not a patient who has had gonorrhoea is free from infection, especially when he desires to marry, is so evident that it needs no emphasis. The difficulty and uncertainty that ordinarily attend the examinations before declaring such patients cured is equally well known to all who are called upon to express an opinion as to the infectiousness of these conditions. Nearly all authorities also agree that the prostate gland and the seminal vesicles are the most frequent sources of recurrent urethral discharges and reinfections. As foci of the dormant gonococcus, they are, therefore, of prime importance, and, in fact, usually the keystone of the situation in the uncertain cases.

The discharge and threads give a basis upon which to work in reaching a conclusion, especially if a discharge is produced with nitrate of silver to increase the number of the gonococci present. The condition of the prostate and vesicles is not determined, however, by this test, nor can we safely trust a negative microscopic examination of the secretion expressed from them as the micro-organisms may be in such small numbers that they will elude a most careful search—the volume of the discharge being too great to allow a smear to be made of it all. Cultures

Read before the Fulton County Medical Society. 1908

taken from it are troublesome and unreliable except in the hands of an expert with a well equipped laboratory.

Palpation of the prostate and vesicles also affords great opportunity of making an error, as considerable induration may exist without gonococci, while a small, apparently normal, organ may contain them.

This maze of uncertainty about a condition with such far-reaching, and sometimes disastrous, results, lead me to try a plan of investigation which is both simple and easy, and when properly carried out, greatly enhances the value of the usual tests. The procedure proposed is to create any irritation of the entire urethral canal by injecting about ʒ1 of a 1-2 to 2 per cent. solution of nitrate of silver with a soft catheter and a syringe attached, or some similar device for placing the medicine in both the anterior and posterior urethra. A few bottles of beer the night before may augment the value of the test by the irritation which usually follows. Three or four hours after the injection when the secretion has become well established, the patient should urinate and the meatus thoroughly dried and sealed with a small amount of collodion or a bit of zinc oxide adhesive plaster. The prostate and vesicles are now massaged and their secretion pressed into the inflamed urethra, where it is left until the patient is forced to void his urine.

As is only too well known, there is no better culture medium for the gonococcus than is the inflamed urethra. Naturally if the expressed secretion, which is prevented from escaping by the collodion at the meatus, contains gonococci they will proliferate when planted upon such fertile soil, even if they were more or less inactive before.

When the patient is compelled to urinate the collodion or adhesive plaster can be removed without much difficulty. This discharge is allowed to escape and the next which forms is collected, and if negative, other smears should be taken 1, 18 or 24 hours later and carefully stained for the gonococcus. If the patient cannot report at suitable times for obtaining the smears, he should be shown how to make them and provided with slides.

In applying the collodion to the meatus the lips should be pressed so that the mucous portion is slightly invaginated and the sides are in close apposition. Two or three coatings will usually be sufficient to keep it sealed. If, however, there is any difficulty, the collodion may be applied over a small pleget of cotton, which reinforces it and makes it more secure. Only a slight burning from the ether is felt if the collodion is used without the cotton, but a little more is experienced when it is used, and may be avoided by soaking a little cotton in a 4 percent. solution of cocaine and placing it over the meatus a few minutes before sealing it. A small strip of adhesive plaster will often prove satisfactory and prevent the escape of the secretion if firmly pressed over the meatus after it has been carefully dried.

The strength of the nitrate of silver to be used must be determined by the previous treatment of the urethra. If active measures have been used a stronger solution is necessary than is required when little treatment has been taken.

This plan of searching for the gonococcus has been tried a number of times and has proved its reliability each time, whether positive or negative. From a review of the literature I have not been able to find where it has been recommended previously. Its simplicity and harmlessness and the fact that it undoubtedly enhances the value of the ordinary test make it worthy of trial.

---

The Pine Ridge Sanitarium for the treatment of throat and lung diseases was formally opened July 10. The location of this institution is ideal and the equipment is all that could be desired—the "cottage tents" being especially commendable. Dr. George Brown and Dr. Louis Rouglin, have the institution under their personal supervision. We believe that such an institution will fill a long felt want and we wish for it a successful career.

---

At the fourth semi-annual meeting of the Chattahoochee Valley Medical Association, Dr. C. L. Williams and Dr. J. H. McDuffie, of Columbus, Ga., read interesting papers.

---

Dr. W. L. Bullard, of Columbus, Ga., has been invited to read a paper at the Pan-American Medical Congress, which convenes at Guatemala in August.

